# An Ultrasensitive Electrochemiluminescent Immunoassay for Aflatoxin M1 in Milk, Based on Extraction by Magnetic Graphene and Detection by Antibody-Labeled CdTe Quantumn Dots-Carbon Nanotubes Nanocomposite

**DOI:** 10.3390/toxins5050865

**Published:** 2013-04-29

**Authors:** Ning Gan, Jing Zhou, Ping Xiong, Futao Hu, Yuting Cao, Tianhua Li, Qianli Jiang

**Affiliations:** 1 The State Key Laboratory Base of Novel Functional Materials and Preparation Science, Faculty of Material Science and Chemical Engineering of Ningbo University, Ningbo 315211, China; E-Mails: hcchcnc@126.com (J.Z.); yongqing1212@gmail.com (P.X.); caoyuting@nbu.edu.cn (Y.C.); litianhua@nbu.edu.cn (T.L.); 2 Faculty of Marine of Ningbo University, Ningbo 315211, China; 3 Department of Hematology, Nanfang Hospital, Southern Medical University, Guangzhou 510515, China; E-Mail: jiangqianlid@yahoo.cn

**Keywords:** aflatoxin M_1_, Fe_3_O_4_-graphene nanocomposite, cadmium telluride-quantumn dots, milk, electrochemiluminescence immunoassay, mycotoxins

## Abstract

An ultrasensitive electrochemiluminescent immunoassay (ECLIA) for aflatoxins M1 (ATM1) in milk using magnetic Fe_3_O_4_-graphene oxides (Fe-GO) as the absorbent and antibody-labeled cadmium telluride quantum dots (CdTe QDs) as the signal tag is presented. Firstly, Fe_3_O_4_ nanoparticles were immobilized on GO to fabricate the magnetic nanocomposites, which were used as absorbent to ATM1. Secondly, aflatoxin M1 antibody (primary antibody, ATM1 Ab1), was attached to the surface of the CdTe QDs-carbon nanotubes nanocomposite to form the signal tag (ATM1 Ab1/CdTe-CNT). The above materials were characterized. The optimal experimental conditions were obtained. Thirdly, Fe-GO was employed for extraction of ATM1 in milk. Results indicated that it can adsorb ATM1 efficiently and selectively within a large extent of pH from 3.0 to 8.0. Adsorption processes reached 95% of the equilibrium within 10 min. Lastly, the ATM1 with a serial of concentrations absorbed on Fe-GO was conjugated with ATM1 Ab1/CdTe-CNT signal tag based on sandwich immunoassay. The immunocomplex can emit a strong ECL signal whose intensity depended linearly on the logarithm of ATM1 concentration from 1.0 to 1.0 × 10^5^ pg/mL, with the detection limit (LOD) of 0.3 pg/mL (*S*/*N* = 3). The method was more sensitive for ATM1 detection compared to the ELISA method. Finally, ten samples of milk were tested based on the immunoassay. The method is fast and requires very little sample preparation, which was suitable for high-throughput screening of mycotoxins in food.

## 1. Introduction

Aflatoxins, which mainly exist in grains, nuts, cotton seeds, as well as some related products, are toxic metabolites and strong carcinogens from a class of fungi (*Aspergillus flavus* and *Aspergillus parasiticus*, e.g.) [1]. Aflatoxin M1 (ATM1), as the hydroxylated metabolite of aflatoxin B1 (ATB1), is usually present in the animal milk contaminated by ATB1. Because of their stronger toxic effects than ATB1 on public health, many governments have provided maximum acceptable limits for residual ATM1 in foodstuffs, especially in milk products [2]. For example, according to Chinese national standards, aflatoxin content cannot exceed 0.5 μg/kg in milk and 5 μg/kg in milk powder, whereas the USA has higher regulations of 500 ng/kg [3]. Thus, the food administration agencies in almost all countries have dedicated much effort to developing sensitive analytical methods for monitoring ultratrace levels of ATM1 (<0.05 μg/kg) in foods [4]. Current strategies for ultrasensitive detection of ATM1 are based mainly on thin-layer chromatography (TLC), high performance liquid chromatography (HPLC) or UV light spectroscopy after extraction and clean-up procedures [3]. These methods are sufficiently sensitive and accurate; however, they often require sophisticated, expensive and heavy instruments that may not be available in laboratories with fewer resources; these methods are especially not fit for mass screening [4]. Therefore, it is urgent to develop affordable, portable and sensitive methodologies for the screening of ATM1 at ultralow concentrations in foodstuffs. 

The immunoassay techniques which are based on the highly specific recognition ability with corresponding antigens by the antibodies have been successfully applied for the detection of aflatoxin [5]. At present, the main immunoassay for ATM1 analysis is carried out using competitive ELISA (cELISA) kits, with confirmation by HPLC (the official method) [6,7]. It is based on a competitive reaction between the free ATM1 in the sample and an ATM1-horseradish peroxidase conjugate, for an immobilized monoclonal antibody for ATM1 on the bottom of 96-well plate monitored by chronoamperometry. The assay is inexpensive and has been used in practical screening analysis. However, it also has some inherent flaws which are difficult to overcome, such as: (1) lower sensitivity compared with HPLC due to the difficulty of determination the pictogram level of ATM1; (2) multiple preparative and washing steps are required to complete the assay, and common time-to-results can take as long as two hours; (3) it also requires a series of laborious sample processing procedures and clean-up to yield reproducible results [8]. Thus, despite their success, there are still pressing needs for ultrasensitive detection strategy of ATM1, especially in the separation and enrichment of analytes from food samples containing highly abundant nonspecific protein and lipid matrixes for point-of-care testing. Recently, there has been an emerging trend of establishing new analytical methods, such as electrochemical and chemiluminescent methods for toxins [7], which not only retains the simplicity, low cost, and portable analytical devices, but also provides new opportunities in the development of precise and sensitive diagnostic devices. Electrochemiluminescent immunoassay (ECLIA) has been proven to be a highly sensitive and selective immunoassay, which combines analytical advantages of electrochemiluminescence (ECL) (sensitive, cost effective, absence of background optical signal, and ease of control by changing electrode potential) with the specificity of immunoassay. Thus ECLIA is suitable for point-of-care determination of ultratrace levels of ATM1 in food. 

The most important step to develop an ultrasensitive ECLIA sensor is to fabricate a signal tag labeled with the antibody of ATM1 (ATM1 Ab) [8]. There have been many reports on the signal tags based on Ru(bpy)_3_^2+^ and luminol [9]. Strongly luminescent semiconductor quantum dots (QDs) (CdS, PbS, CdTe and ZnS) have found potential applications in biological imaging and labeling, which also have unique advantages such as excellent opto-electronic properties [10,11]. Among the compound semiconductors, water-soluble CdTe has attracted growing interests on versatility in surface modification with various biomolecules [12]. Although ECLIA analysis based on QDs has many advantages, the reports concerning the detection of toxins with QDs ECL are relatively scarce. The reasons are partially because that the ECL of semiconductor QDs is weaker than that of conventional luminescent reagents such as luminal or Ru(bpy)_3_^2+^. It is reported that carbon nanotube (CNT) can be used to enhance the ECL of CdS QDs film by reducing the injection barrier of electrons to the QDs [13]. Moreover, Wang and Mountziaris [14] have reported on immunoassays employing ZnSe QDs conjugated to proteins to form QD-based biomolecular sensors. This phenomenon enables the development of homogeneous, separation-free immunoassays for rapid quantitative detection of proteins in solution. The development of the QDs-CNT compound requires conjugation of both ingredients through polymer. Poly(diallyldimethylammonium chloride) PDDA is a strong candidate for further modification to develop nanocomposites [14]. With readily available free amine groups, this polymer offers more uniform coating of the carbon nanotubes, thus improving the solubility of CNTs, and reducing the potential toxic effects if used on a labeling antibody. Moreover, a single CNT can encapsulate dozens of CdTe QDs (as the signal source) to produce amplification of an ECL signal. Moreover, the surface of the hybrid particles can also be labeled with attached antibodies as tags. Taking into consideration the above advantages, the CdTe QDs-CNTs composites were fabricated and employed for immobilizing ATM1 monoclonal antibody (ATM1 Ab1) on it in the article. Thus a novel signal tag (ATM1 Ab1/CdTe QDs-CNTs) for ECL biosensors was synthesized. 

**Scheme 1:**
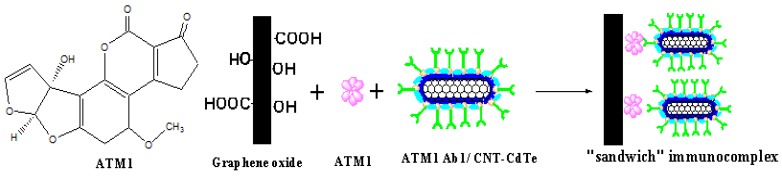
The “sandwich” immunoassay for detection of ATM1.

Another key issue for the ECLIA sensor is to choose a convenient method for immobilizing the signal probe on the electrode and maintain the biocompatibility of the antibody to greatest extent. However, it is very difficult to achieve the aim, because whatever the chemical grafting or physical adsorption method employed, it can inevitably generate deactivation to antibody and reduce its identification ability for antigen. And once the antigen is conjugated with the antibody, it is difficult for the electrode to be reused. Moreover, the antibodies are very expensive. Thus, if a kind of material with recognition feature for ATM1 can be utilized to immobilize on the electrode instead of the antibody, a label-free ECL electrode would be obtained, which will greatly reduce the cost and simplify the fabrication process. Graphene oxides (GO), as one kind of flat monolayer of carbon atoms tightly packed into a two-dimensional honeycomb lattice, has come to the forefront of research in electrochemical sensors recently, because of its unique electronic properties and high electrical conductivity [15,16]. Furthermore, GO can also been served as an extraordinarily wonderful adsorbent material for enriching aromatic organic compounds in a complex matrix [17]. Because ATM1 is a compound with a large aromatic ring, GO will thus be used to absorb ATM1 and separate the ATM1 from the tested samples in this paper. Furthermore, the ATM1 Ab1/CdTe-CNT signal tag can be employed to conjugate with the ATM1 absorbed on GO to form a “sandwich” immunocomplex (GO/ATM1/ATM1 Ab1/CdTe-CNT), which can generate ECL signal on the electrode. Certainly, other aromatic ring-containing substances can also be adsorbed on the surface of GO. However, the signal tag labeled with ATM1 Ab1, cannot react with other absorbed matter other than ATM1. Thus the ECL signal can only reflect the amount of ATM1 enriched on GO. The “sandwich” detection scheme (Scheme 1) is significantly different from the conventional competitive enzyme immunoassay (cELISA) for toxin. Firstly, when the concentration of ATM1 is increased, the proposed method will generate an enhanced detection signal, while the signals in cELISA decrease, because less enzyme-labeled antigen as competitive substances would bind to the bottom of the microplate, which can catalyze the substrate to produce the signal. Usually, most of the interference substances (such as protein and lipid) in food samples have high resistance, which can decrease the electrochemical signal. Therefore, the new immunoassay results can better reflect the real content of the analyte. Secondly, the scheme is similar to double-antibody sandwich reaction with two recognition sites (one from GO, and the other from the antibody on the signal tag), which will have higher recognition ability for ATM1 than competitive ELISA with only one recognition site. To the best of our knowledge, no report about establishing ECL immunoassay on the above “sandwich” scheme has been published. 

In recent years, magnetic separation technology is a useful and convenient tool that has recently been considered in sample treatment and separation from complex background in foodstuff [18]. The magnetic component participation allows for a controllable rebinding process that may offer a rapid and simple alternative to conventional centrifugation and filtration [19]. For instance, graphene loaded with magnetic materials could realize the retrieval and separation of graphene from dispersion rapidly and effectively. Moreover, at the same time maintain the intrinsic properties of graphene at extreme [19]. Nanometer-sized magnetic particles of iron (Fe_3_O_4_ NPs), are potential candidates in magnetic separation and biomedical applications [20]. Thus, the hybrid material combining magnetic Fe_3_O_4_ NPs and graphene absorbent has the potential to provide a simple, flexible, and highly selective pretreated method that may be much more practical than the complicated centrifugation procedure employed in extraction of ATM1 in food samples. Herein, the magnetic GO (Fe-GO) was prepared as absorbent for ATM1. It also has other advantages: firstly, after ATM1 was extracted by Fe-GO, the complex can be easily modified on the surface of electrode by a magnet. Secondly, GO with high conductivity can also accelerate the electron transfer speed, which would amplify the ECL signal [21,22]. Thirdly, the magnetic probes can be easily immobilized on the surface of the screen printed carbon electrode (SPCE) after adding a magnet on the bottom of the plane electrode [23].

In this work, we report a novel ultrasensitive “sandwich” immunoassay scheme for ATM1, the immunocomplex was conjugated among magnetic Fe-GO absorbent, the free ATM1 in the sample and the immobilized monoclonal antibody from CdTe-CNT signal tag based on a SPCE electrode. The magnetic hybrid particles (Fe-GO), by the aid of high affinity graphene with aromatic pollutants, thereby assure the high selective adsorption of ATM1. Moreover, it can be separated easily after extracting ATM1 in food samples by external magnetic field, and avoid the interference of complex matrix. The novel signal probe (ATM1 Ab1/CdTe QDs-CNTs) can greatly enhance the ECL signal of the immunosensor. The assay was employed to detect ATM1 in real milk samples and a detection limit of 0.3 pg/mL was achieved. The immunosensor exhibited many functions in simple instrumentation, high sensitivity, disposable, magnetic separation and enrichment, which may find promising applications for toxin analysis in food.

## 2. Results and Discussion

### 2.1. Characterization of Sorbents

**Figure 1:**
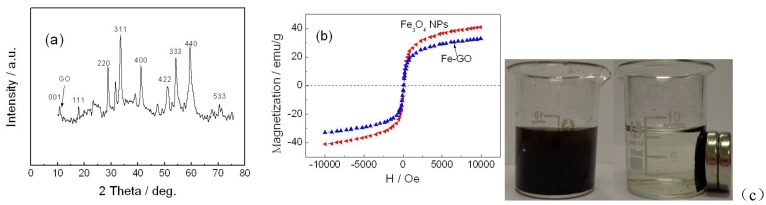
The (**a**) XRD; (**b**) magnetization hysteresis loops spectrum and (**c**) magnetic separation of Fe-GO before and after adding the magnetic field.

The XRD pattern of the Fe-GO was obtained in Figure 1a. The appearance of the diffraction peak at 2θ = 10.3° (001) could be ascribed to the reflection of the GO. Six diffraction lines are observed in the representative XRD pattern of Fe_3_O_4_ at 2θ = 30.1°, 35.4°, 43.3°, 54.5°, 57.3° and 62.8°. These diffraction lines can be assigned to the (220), (311), (400), (422), (511) and (440) reflections, respectively, in the cubic spinel crystal structure of Fe_3_O_4_ with cell constant α = 8.397 Å (JCPDS card No.19-0629). From the XRD pattern of Fe-GO analysis, the main characteristic peaks of Fe_3_O_4_ and GO are located at 10.3°, 30.1°, 35.4°, 43.3°, 54.5°, 57.3°and 62.8°, indicating that the product is composed of two phases: Fe_3_O_4_ and GO. 

The magnetic property of the Fe-GO nanocomposite is investigated by VSM (Figure 1b). Maximum saturation supermagnetizations of Fe_3_O_4_ and Fe-GO are measured at 38.27 and 31.85 emu/g, respectively. Although the addition of the nonmagnetic portion leads to decreased saturation supermagnetizations, the obtained Fe-GO still have a high saturation supermagnetization of 31.85 emu/g. According to Ma’s study, a saturation supermagnetization of 16.3 emu/g is enough for magnetic separation from solution with a magnet [24]. The magnetic separation was achieved before and after adding the magnetic field (Figure 1c). The results implied that Fe-GO sorbent can be dispersed into water solution readily. Once the external magnetic field is taken away, these sorbents can re-disperse rapidly.

**Figure 2:**
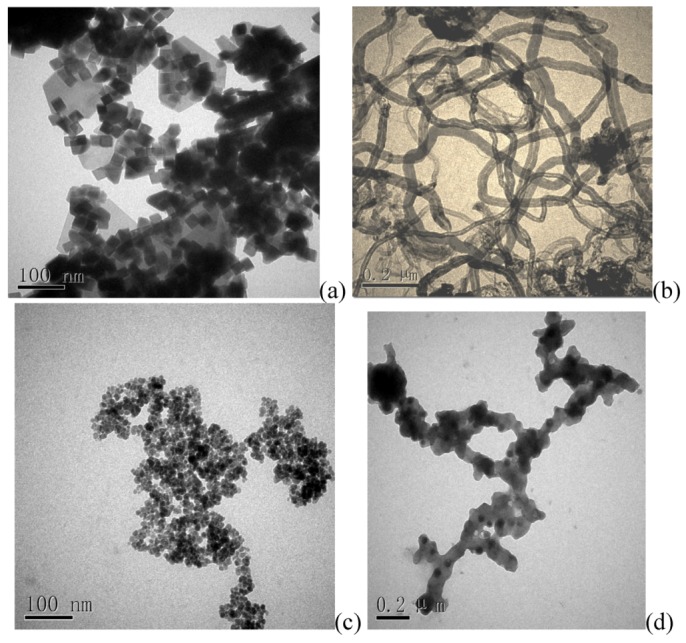
The TEM of (**a**) Fe-GO; (**b**) CNT; (**c**) CdTe QDs and (**d**) CdTe-CNT nanocomposite.

The microstructure transformations and differences of Fe-GO, CNT, CdTe QDs and CdTe-CNT nanocomposite can be observed by transmit electron microscopy (TEM) images (Figure 2). As to exfoliated Fe-GO (Figure 2a), large sheets (a few hundred square nanometers) of GO were observed. They were transparent and entangled with each other. Figure 2a also showed that the Fe_3_O_4_ particles were uniformly grafted and dispersed on graphene oxide sheets. We can see that the small Fe_3_O_4_ particles are in isometric form. Figure 2b shows the CNT have long tube shape. Figure 2c shows the TEM images of (CdTe QDs). They appear quasi-spherical in shape with an average diameter of about 10 nm. Figure 2d demonstrated that QDs were concentrated around the CNT, which visually confirmed that the successful attachment has taken place. The BET surface area of Fe-GO and CdTe-CNT is 142.8 and 138.5 m^2^/g, respectively.

### 2.2. The Fluorescence of CdTe-CNT Nanocomposite

**Figure 3:**
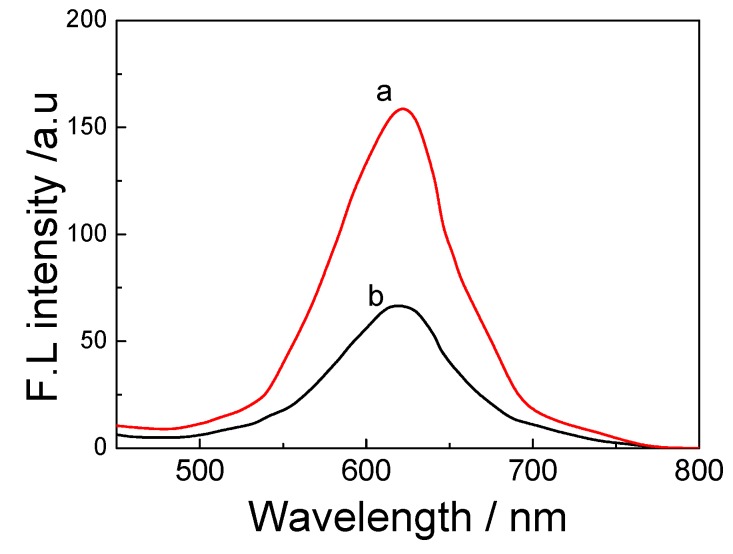
Fluorescence spectrum of (**a**) free CdTe and (**b**) CdTe-CNT.

The CdTe-CNT complex consists of covalently bound ingredients, therefore, fluorescence spectroscopy was performed to investigate CNT photo bleaching effects on QDs. The CdTe QDs have a distinct emission spectrum peak at 620 nm (Figure 3, curve a). The –COOH functionalized CNTs alone failed to emit a signal. When CNT was covalently bounded to QDs through the attachment of PDDA, the emission peak at 620 nm reduced (Figure 3, curve b).

### 2.3. Kinetics Analysis

**Figure 4:**
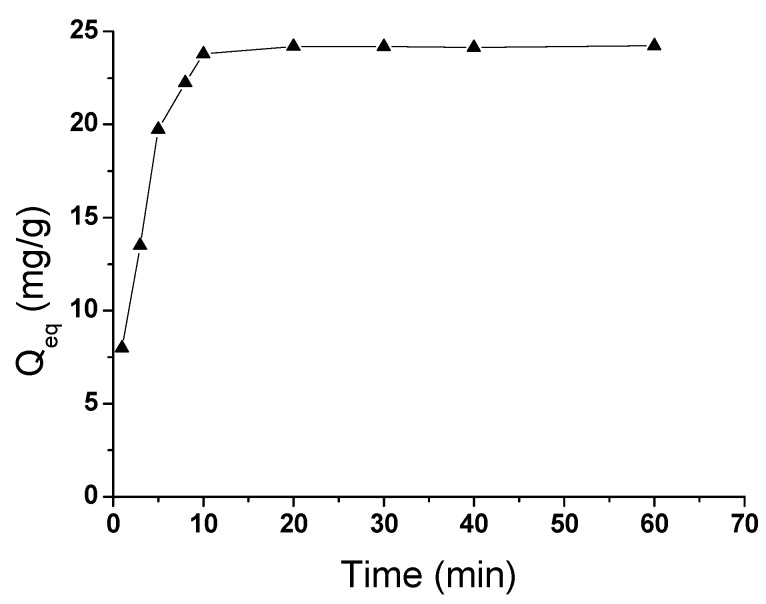
The effect of adsorption time on the adsorption capacity.

To investigate the adsorption kinetics of ATM1 onto Fe-GO, the Fe-GO was exposed to ATM1 solution (10 mL 0.25 mg/L) in the range of 5–60 min at the optimum pH (7.4) and adsorbent dosage (1.5 mg/mL). The results (Figure 4) showed that the rate of adsorption for ATM1 was very rapid at the first 10 min (reached to 95% of the equilibrium); thereafter, it increased gradually and reached a plateau, indicating the equilibrium of the system. The trend of adsorption kinetics was due to the adsorption of ATM1 on the exterior surface of adsorbent at the initial period of contact time. When the adsorption on the exterior surface reached saturation point, the ATM1 diffused into the pores of the adsorbent and was adsorbed by the interior surface of the adsorbent. 

In order to show the most suitable model for the experimental data, two different kinetic models were used in this study. They are pseudo-first-order rate model and pseudo second-order rate model. Pseudo-first-order rate equation is expressed as follows Equation (1):

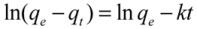
(1)
Where *q*_e_ is the amount of ATM1 (mg/g) adsorbed per unit mass of adsorbent at equilibrium, *q*_t_ is the amount of ATM1 (mg/g) adsorbed at time *t* (min^−1^), and *k* is the equilibrium rate constant of pseudo first-order. The pseudo second-order model can be expressed as Equation (2):

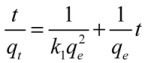
(2)
Where *k*_1_ (g mg^−1^ min^−1^) is the pseudo second-order rate constant. Based on the two models, curve fitting was performed and the parameters in the models and regression coefficients (*R*^2^) for the two kinetic models were obtained. The Lagergren-first-order rate constant *k* and *Q*_1_ can be determined from the intercept and slope of the plot obtained by plotting Ln(*Q*_eq_− *Q*_t_) *versus* t; the Pseudo-second-order rate constant *k*_1_ and *Q*_2_ can be determined from the intercept and slope of the plot obtained by plotting *t*/*Q*_t_ *versus t*. The calculated parameters for Lagergren-first-order model and Pseudo-second-order model and the correlation coefficients (*r*^2^) are listed in Table 1.

**Table 1:** Kinetic parameters for adsorption of cationic ATM1 onto Fe-GO.

Sample	*Q*_eq _(mg/g)	Lagergren-first-order model			Pseudo-second-order model		
		*Q*_1_ (mg/g)	*k* (min^−1^)	*r*^2^	*Q*_2_ (mg/g)	*k*_1_ (g mg^−1^ min^−1^)	*r*^2^
ATM1	23.2	22.5	0.354	0.992	23.8	0.21	0.994

Comparison of *Q*_eq_, Table 1 showed that the calculated *Q*_2_ values of the Pseudo-second-order equation are generally closer to the experimental *Q*_eq_ values compared to the calculated *Q*_1_ values of Lagergren-first-order equation. Also the correlation coefficients for the second order kinetic model obtained at all the studied ATM1 were above 0.992. Thus, the adsorption process studied followed the Pseudo-second-order kinetic model better. 

### 2.4. Adsorption Isotherms

Adsorption isotherm is important to understand the sorption properties of ATM1 on Fe-GO. Several adsorption isotherm equations are available and the two important isotherms are selected in this study, the Langmuir and Freundlich isotherms. ATM1 adsorption isotherms were determined at the initial pH 7.4. 1.5 mg/mL of adsorbent was added into 200 mL beaker with a definite volume of 10 mL each with different ATM1 concentrations ranging for 0.1 mg/mL to 0.25 mg/mL. After shaking for 20 min, the Fe-GO was removed from the solution by magnetic separation and the concentration of the dye in the resultant solution was analyzed.

The widely used Langmuir isotherm assumes that sorption takes place at specific homogeneous sites within the adsorbent, and has been successfully applied to many sorption processes. The linear form of Langmuir isotherm is given by the following equation:

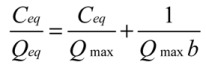
(3)
Where *C*_eq_ (mg/L) is the equilibrium concentration, *Q*_eq_ (mg/g) is the amount adsorbed at equilibrium, *Q*_max_ (mg/g) is the maximum adsorption capacity and b (Langmuir constant, L/mg) is the energy of adsorption. 

The Freundlich isotherm describes the equilibrium on a heterogeneous surface. Therefore, it was regarded as a physical adsorption combined with the effect of chemical adsorption. The isotherm is described by the following equations:

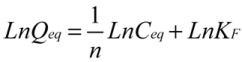
(4)
Where *K*_F_ and *n* are Freundlich characteristic constants, indicating the adsorption capacity and the adsorption intensity, respectively. It has been shown that n values from 2 to 10 represent good adsorption potential of the adsorbent.

**Figure 5:**
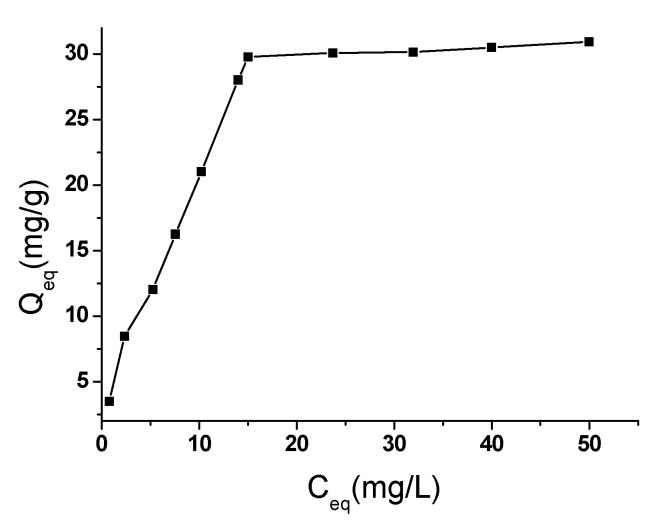
Adsorption isotherms of ATM1 onto Fe-GO.

Figure 5 shows the adsorption isotherms for ATM1. The calculated parameters for Langmuir and Freundlich isotherms and the correlation coefficients (*r*^2^) are listed in Table 2. The correlation coefficients indicate that Langmuir isotherm has been fitted better for the adsorption of the ATM1 on the Fe-GO. It indicated that the cationic ATM1 could be adsorbed on the Fe-GO as a monolayer adsorption. The n and *Q*_max_ values suggest that the ATM1 could be easily to be adsorbed on the Fe-GO and the Fe-GO absorbent exhibited a high adsorption capacity for the ATM1. 

**Table 2:** Adsorption isotherms parameters of cationic ATM1 onto GO-Fe.

	Langmuir model			Freundlich model		
	*Q*_max _(mg/g)	*B* (L/mg)	*r*^2^	*K*_F _(mg/g)	*n*	*r*^2^
ATM1	29.4	0.093	0.993	37.8	2.31	0.992

### 2.5. Optimization of pH for Extraction

The pH of solution can play an important role for the adsorption of the analyte by affecting both the existing forms of the target compounds and the charge species and density on the absorbent surface. The experiments were carried out using 0.25 mg/L ATM1 solution containing various pH ranging from 3.0 to 10.0.The results showed that the sorption percentage of ATM1 on Fe-GO fluctuates very little in pH range of 3–8, which suggests that Fe-GO are excellent adsorbents for ATM1’s removal from large volumes of aqueous solutions. When the pH is greater than 8, the sorption percentage of ATM1 on Fe-GO clearly decreases. This can be ascribed to the fact that more oxygen containing groups (such as −COOH and −OH) on Fe-GO surfaces are ionized (carrying negative charge) at high pH values. More water molecules then prevent ATM1 from getting closed to the adsorbent. 

### 2.6. ECL Characterization of CdTe, CdTe-CNT and ATM1 Ab1/CdTe-CNT Signal Tag

**Figure 6:**
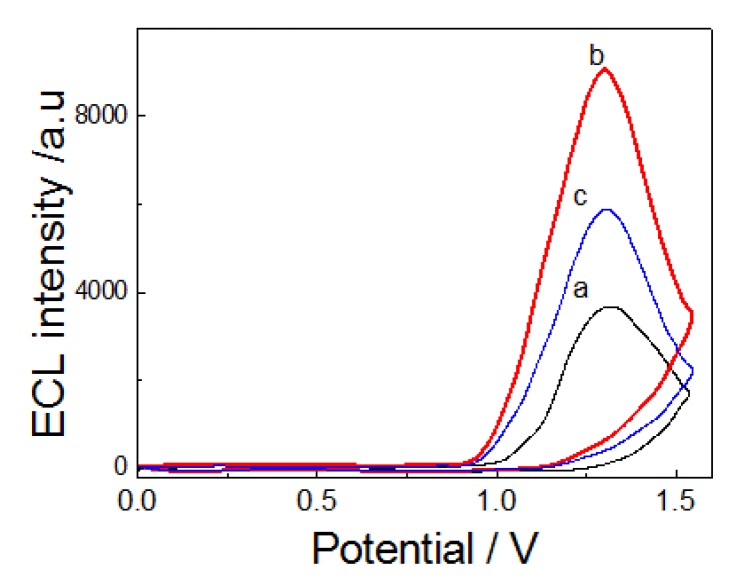
ECL-potential curves of (**a**) CdTe QDs; (**b**) CdTe-CNT and (**c**) ATM1 Ab1/CdTe-CNT bioconjugates modified SPCE electrode at 0.1 mol/L PBS (pH 7.4) containing 0.1 mol/L KCl and 0.1 mol/L K_2_S_2_O_8_. Scan rate: 100 mV/s. The voltage of the PMT was set at 600 V.

Figure 6 showed the ECL-potential curve of CdTe QDs, which could generate weak ECL, but the ECL intensity of CdTe-CNT composite film (curve b) on SPCE was much higher than that of CdTe QDs (curve a), suggesting that CNT have better electric conductivity and more specific surface area to facilitate the ECL reaction, which was more favorable for fabricating an ultrasensitive ECL immunosensor. Curve c showed that the ATM1 Ab1/CdTe-CNT bioconjugates can also emit the ECL signal, while its intensity was lower than CdTe-CNT, which suggested ATM1 Ab1 hindered the electron transfer between CdTe-CNT and SPCE electrode. ATM1 Ab1/CdTe-CNT bioconjugates can act as signal tag for detecting ATM1. The reaction equation is shown as the following:

CdTe + e^− ^→ CdTe^−^ (1)

S_2_O_8_^2−^ +e^− ^→ SO_4_^2−^ +SO_4_^−^ (2)

CdTe^−^ + SO_4_^−^→ CdTe* +SO_4_^2−^ (3)

CdTe* → CdTe+hυ (4)

### 2.7. Characterization of the ECL Immunosensor

#### 2.7.1. ECL Behavior and Amplification Effect

**Figure 7:**
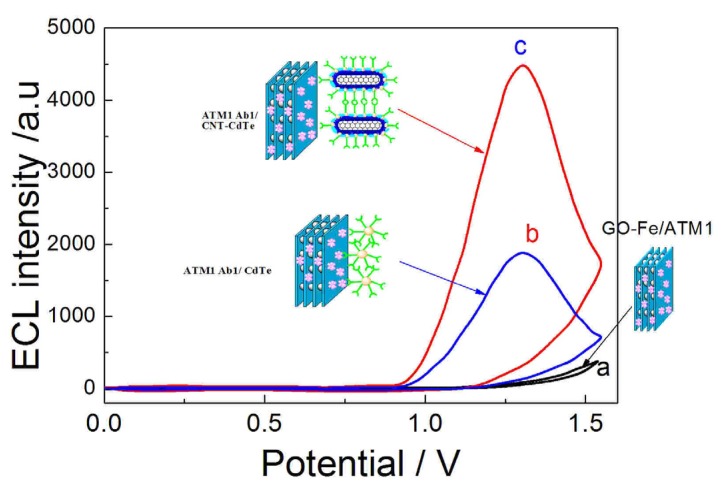
ECL-potential curves of the (**a**) Fe-GO; (**b**) Fe-GO/ATM1/ATM1 Ab1/CdTe; (**c**) Fe-GO/ATM1/ATM1 Ab1/CdTe-CNT sandwich immunocomplex modified SPCE electrode. Other conditions are the same as Figure 6.

The fabrication process of the ECL immunosensor was monitored by measuring the ECL signals after each immobilization step (Figure 7). It could be seen that there was no ECL signal for Fe-GO after it absorbs the ATM1(curve a), whereas there was an obvious enhancement of ECL intensity after a sandwich immunoreaction between Fe-GO, 5 pg/mL ATM1 and the antibody on the signal tag (ATM1 Ab1/CdTe) (curve b). There was a 2.5-fold enhancements in ECL signal for ATM1 detection by ATM1 Ab1/CdTe-CNT tag (curve c) when comparing with CdTe as the signal tag matrix (curve b). All these imply that the CNT can greatly accelerate the electron transfer between CdTe QDs and SPCE electrode, and then obviously amplify the ECL signal. 

#### 2.7.2. Optimization of Immunoassay Conditions

**Figure 8:**
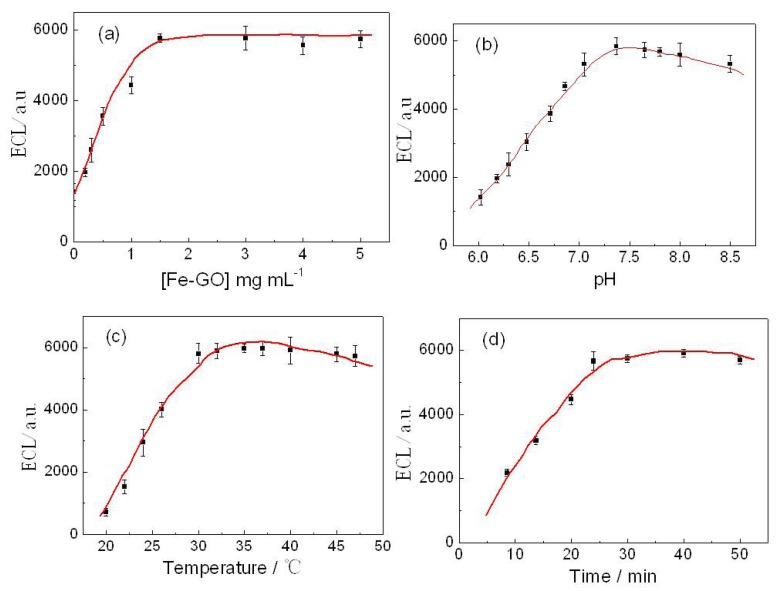
Effect of (**a**) Fe-GO composite solutions, (**b**) pH, (**c**) incubation temperature, and (**d**) time on the ECL intensity the immunosensor toward 5 pg/mL ATM1. Other conditions are the same as Figure 6.

Figure 8 showed the effects of different immunoassay operating factors, such as the amount of the Fe-GO composite solutions absorbed on the electrode (curve a), pH of the supporting electrolyte (curve b), incubation temperature (curve c), and incubation time (curve d), on the corresponding ECL response curves. The concentration of Fe-GO highly influenced the performance of the ECL signal response. Concentration of the probes solution (0.2, 0.3, 0.5, 1, 1.5, 3.0, 4.0, and 5.0 mg/mL) was chosen for experiments in Figure 8a. Results revealed that 1.5 mg/mL Fe-GO solution was optimal. Further, based on the experimental results, 10 µL of capture probes solution was selected as the optimal dosing volume on the electrode surface, which could be easily absorbed on the surface of electrode by magnet. The pH of the background solution could greatly affect the ECL response of the immunosensor, because the activity of the antibody protein might be influenced by the acidity of the solution. Thus, the effect of pH from 6.0 to 8.5 on the immunosensor performance was investigated using 5 pg/mL ATM1 solutions. As shown in Figure 8b, the maximum ECL intensity could be obtained at pH 7.4 by the immunosensor. Thus, the detection was performed in pH 7.4 PBS throughout the experiment. The effect of incubation temperature and time on the ECL response of the immunosensor was also investigated. It could be seen from Figure 8c that the ECL signal first increased and then reduced with the increase of incubation temperature from 20 °C to 50 °C and a maximum ∆ *I*_ECL_ was obtained at 37 °C. Figure 8d showed that the ECL signal increased with the increase of incubation time and reached a plateau at 25 min. Therefore, 37 °C and 25 min were selected as the optimum incubation temperature and time in this study.

### 2.8. Analytical Performance

**Figure 9:**
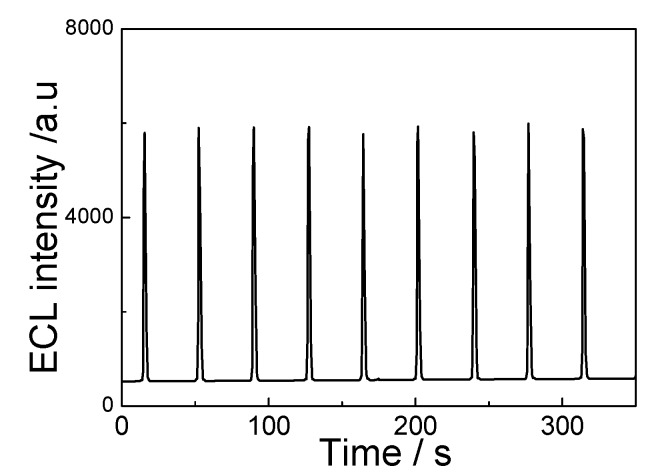
ECL emissions from the immunosensor to the final immunocomplex using 5 pg/mL ATM1 solution under continuous potential scanning for nine cycles. Scan rate: 100 mV/s.

As shown in Figure 9, when consecutive scans from 0 to −1.6 V were performed for nine cycles, no obvious change of the ECL intensity of the immunosensor curve using 5 pg/mL ATM1 solution was observed. The ECL signals were strong and stable, suggesting that the immunosensor was suitable for ECL detection.

**Figure 10:**
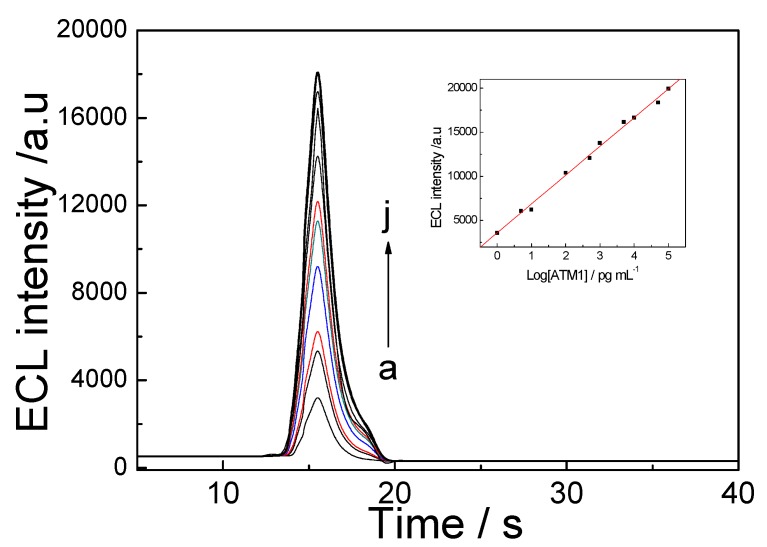
ECL profiles of the immunosensor before (**a**) and after (**b**–**j**) incubating in different concentrations of ATM1 in pH 7.4 PBS containing 0.1 mol/L KCl and 0.1 mol/L K_2_S_2_O_8_. ATM1 concentration (pg/mL): (**a**) 0; (**b**) 5; (**c**) 10; (**d**) 100; (**e**) 5.0 × 10^2^; (**f**) 1.0 × 10^3^; (**g**) 5.0 × 10^3^; (**h**) 1.0 × 10^4^; (**i**) 5.0 × 10^4^;.(**j**) 1.0 × 10^5^, Inset A: linear plots of ECL intensity *vs*. log (ATM1) concentrations.

Under the optimal conditions, the ECL immunosensor was carried out to analyze various concentrations of ATM1 standard solution, and the ECL response was recorded (Figure 10). The intensity of the ECL showed linear dependence of the logarithm of ATM1 concentration in the range from 1.0 pg/mL to 100 ng/mL and the linear regression equation was *I* = 3520.3 + 1724 log(ATM1), with a coefficient of 0.9965. The limit of detection (LOD) was 0.3 pg/mL (*S*/*N* = 3).

### 2.9. Specificity, Reproducibility and Stability of the ECL Immunosensor

To investigate the specificity of the immunosensor, we mixed 1, 10, 100 pg/mL ATM1, respectively, with 35 g/L casein and 20 g/L low-density lipid (LDL), and then detected the ECL response of the mixture. Compared with the ECL response of the immunosensor in 1, 10, 100 pg/mL ATM1, respectively, no significant difference was observed with the R.S.D changed between 1.2% and 3.2%, thereby indicating that the usual matrix substance of casein and LDL in milk powder could not cause the observable interference. The LOD for detection of ATM1 in these matrices was 0.3 pg/mL which is consistent to the LOD listed in Section 2.4. The results suggest that the immunosensor displays good specificity for the determination of ATM1 in milk. 

After the immunosensor was stored in pH 7.4 PBS at 4 °C over 30 days, it was used to detect the same ATM1 concentration, the analytical performances did not show an obvious decline, demonstrating that the immunosensor had good stability. The reproducibility of the immunosensor was evaluated by detecting 5 pg/mL ATM1 (6 times) by the same SPCE electrode. The relative standard deviation (RSD) of the measurements was 3.5%, indicating the excellent reproducibility of the immunosensor.

Regeneration of the immunosensor was achieved by removing magnet at the back of the SPCE, and washing away the magnetic immunocomplex with pH 7.4 PBS solutions for six times. Subsequently, the renewed SPCE was reused to detect 5 pg/mL ATM1 again by adding new magnetic Fe-GO/ATM/signal tag immunocomplex. The consecutive regeneration were repeated six times, an average recovery of 95.4% and an intra-assay RSD of 2.3% were acquired, demonstrating that the proposed immunosensor could be regenerated simply by removing the exerted magnetic field.

After the immunosensor was stored at 4 °C over two weeks, it was used to detect the same ATM1 concentration. The response of the immunosensor retained about 95% of its initial value, demonstrating that the immunosensor had a good stability. Thus, the developed immunosensor is an appropriate tool for the detection of ATM1 based on the obtained results (data not shown).

### 2.10. Application of the Immunosensor in Milk Samples

**Figure 11:**
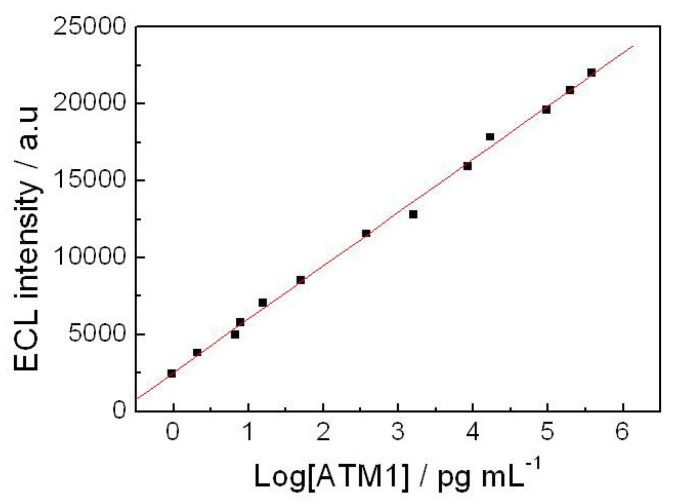
The standard curve of ECL intensity *vs*. log (ATM1) concentrations in milk samples.

In order to investigate the applicability and reliability of the prepared ECL immunosensor for applications, recovery experiments were performed by standard addition methods in milk samples spiked with ATM1. In order to obtain the LOD and concentration of ATM1 in real milk samples, a standard curve along with a limit of detection in milk was obtained in Figure 11. The linear regression equation was *I* = 3012.3 + 1802 log(ATM1), with a coefficient of 0.9972. The limit of detection (LOD) was 0.3 pg/mL (*S*/*N* = 3). Results, as listed in Table 3, showed an acceptable recovery in the range of 90%~120% and CV was from 1.4% to 3.1%. The method was also compared with the routine ELISA method. The results are consistent between each other, which also revealed that the developed ECL immunosensor may provide an efficient tool for ultrasensitive determination of ATM1 in milk samples. 

**Table 3:** Recovery tests for ATM1 in spiked milk samples(*n* = 3).

Sample	Added (μg/kg)	Found by the method (μg/kg)	Found by ELISA	Recovery (%)	CV (%)
1	0.01	0.009	ND	90	0.75
2	0.05	0.046	ND	92	2.3
3	0.1	0.095	0.12	95	1.4
4	0.2	0.2	0.23	100	2.5
5	0.5	0.6	0.47	120	2.7
6	1.0	1.13	1.12	113	3.1

* ND: not detected.

## 3. Experimental Section

### 3.1. Materials and Reagents

Graphene oxide (purity 99.9995%) and single carbon nanotube were obtained from Aladdin Chemistry Co. Ltd. (Shanghai, China). Aflatotoxin M1 (ATM1) was purchased from sigma Co. Ltd. (St. Louis, MO, USA). CdTe was synthesized according literature [12]. Milk samples were obtained from local supermarkets. The monoclonal antibodies against ATM1 (MAb) were purchased from Maine Biotechnology Services (Meridian Life Science, Inc., Memphis, TN, USA). Casein, bovine albumin (BSA), Tween20, potassium permanganate (KMnO_4_, 98 wt%), sulfuric acid (H_2_SO_4_, 98 wt%), phosphorus pentoxide (P_2_O_5_, 99 wt%), hydrogen peroxide (H_2_O_2_, 30 wt%), hydrochloric acid (HCl, 37 wt%) and potassium peroxydisulfate (K_2_S_2_O_8_, 99 wt%) were obtained from Shanghai Chemical Reagent Co. Ltd. (Shanghai, China). All reagents were of analytical grade and were prepared using deionized water.

### 3.2. Apparatus

A model 550 microplate reader (Bio-Rad Laboratories, Shanghai, China) was used to read the absorbance on ELISA plates at 655 nm. ECL signals were measured with a MPI-A multifunctional electrochemical and chemiluminescent analytical system (Remax Electronic Instrument Limited Co., Xi’an, China, 350 nm–650 nm) by a conventional three-electrode configuration at room temperature. SPCE (3 mm diameter) was used as a working electrode; an SCE and a Pt wire were used as a reference and auxiliary electrodes, respectively. The spectral width of the photomultiplier tube (PMT) was 200–800 nm and the voltage of the PMT was 500–800 V in the detection process. Transmission electron microscopy (TEM, Hitachi, Tokyo, Japan). Fluorescence spectrometer (Hitachi, Tokyo, Japan). X-ray diffraction (XRD, Bruker D8 Focus, Breman, Germany). Ultrasonic cleaner (Kudos, Shanghai, China). Supercentrifuge (Anting, Shanghai, China).

### 3.3. Synthesis of CdTe-CNT QDs Conjugates

Three mg of single-walled carbon nanotubes (CNT) were mixed with 1.5 mg EDC and 0.9 mg NHS, dissolved in 3 mL of DMF, and stirred at room temperature for 2 h. Then the material was ultrasonicated for 3 min at room temperature, before 3 mg of PDDA was dissolved in 1 mL of 1 mol/L NaOH. CNT and PDDA solutions were mixed together and stirred at room temperature for 1 h. The mixture was then taken and centrifuged at room temperature for 15 min at 5000 rpm. Distilled water was added to precipitate, and the centrifuge cycle repeated. The final precipitate was oven-dried for 10 min at 68 °C, and the dried weight calculated. PBS was then added to obtain 1 mg/mL concentration. 

CdTe QDs with free surface –COOH groups was mixed with methanol at 1:1 volume ratio, and centrifuged 5000 rpm at room temperature for 10 min. Supernatant was then discarded, and 1 mg/mL concentration of CdTe QDs solution was prepared. CdTe QDs and CNT-PDDA solutions were combined to make up final solution of 1:1 concentration ratio. Thus the CdTe-CNT conjugate was obtained.

### 3.4. Antibody-Labeling CdTe-CNT QDs Conjugate (ATM1 Ab1/CdTe-CNT)

**Figure 12:**
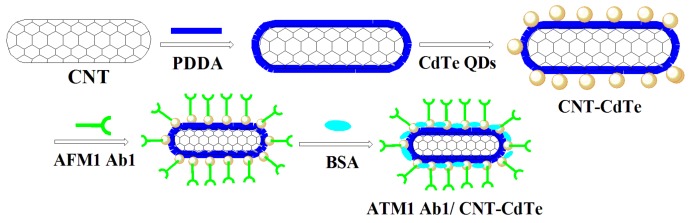
The synthesis steps for ATM Ab1/CdTe-CNT signal tag.

Ten µL of ATM1 monoantibody (ATM1 Ab1) concentrates was mixed with 1 mL of CdTe-CNT complex; 5 mg of NHS and 5 mg EDC was added, and shaken for 20 min at room temperature. One hundred µL of diluted antibody solution and 5% BSA, which was employed for coating the active sites of CdTe QDs, were placed in a shaker at speed 30 for 10 min at room temperature. Mixtures were then centrifuged using 100 kDa filter system at room temperature for 10 min at 5000 rpm, before the supernatant was discarded. The ATM Ab1/CdTe-CNT signal tag can be acquired (Figure 12).

### 3.5. Synthesis of Fe-GO

The magnetic graphene oxide-Fe_3_O_4_ nanoparticles hybrid (Fe-GO) was synthesized by the *in situ* chemical co-precipitation of Fe^2+^ and Fe^3+^ in an alkaline solution in the presence of GO according to the literature [25]. 

### 3.6. The Sandwich ECLA Procedure for Detection of ATM1 in Milk Samples

Milk samples were purchased from a grocery store. A series of milk samples spiked with different concentrations of ATM1 from 0.01 to 1.0 μg/mL were prepared. Each sample was diluted with PBS to 10 mL. The preparation of the sample consisted of centrifugation for defatting for 15 min at 6000 rpm. After centrifugation, the phases were completely separated into layers of fat, cream and skimmed milk from top to bottom, respectively. The defatted sample was tested directly. 

**Figure 13:**
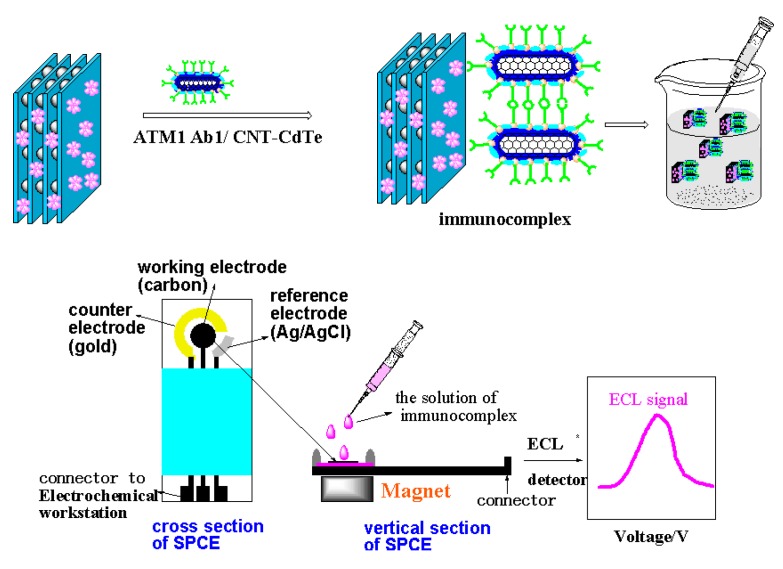
The detection of ATM1 in samples by the sandwich immunoassay.

Different amounts of milk samples were then introduced into the tubes containing 300 µL of the 1.5 mg/mL Fe-GO, The mixture was incubated for 10 min at room temperature. When the reaction was over and the Fe-GO/ATM1 conjugation was formed, 200 µL of the ATM1 Ab1/CdTe-CNT was added to the above tube and incubated for another 25 min at 37 °C and then the incubation sandwich-type complex was formed. The mixture was magnetically purified for 5 min, and the precipitate was washed twice in a high-gradient magnetic field using pH 7.4 PBS buffer solution. Finally, the solution of sandwich immunocomplex was added drop-wise to the electrode’s surface, whose back was placed with a magnet in advance. The electrodes were scanned from 0 to −1.6 V with a scan rate of 100 mV/s, and ECL signals were measured (Figure 13). After each detection, the electrode was instantaneously washed with PBS solution 5 times to remove the immunocomplex after removing the magnet. Standard curves were obtained using 1–10^5^ pg/mL ATM1 for electrochemical immunoassay.

### 3.7. Material Characterization

The particle size and structure of the sorbents were characterized by a transmission electron microscope (TEM, JEOL 2100). Magnetic property was analyzed using a vibrating sample magnetometer (VSM, LDJ9600). The XRD characterization was performed using X-ray diffraction (Bruker D8 Focus, Breman, Germany) with Cu Kα radiation at room temperature. 

## 4. Conclusions

In the present work, an ultrasensitive ECL immunosensor for ATM1 in milk, based on the sandwich conjunction reaction between the Fe-GO by using different concentrations of ATM1 and CNT/QDs as the signal tag was constructed. The CdTe-CNT nanocomposite exhibited high ECL intensity and stability. Moreover, the magnetic composite (Fe-GO) has good adsorption capacity for ATM1, which can enrich ultratrace levels of ATM1 and easily separate them from complex background in foodstuffs through magnetic separation. We confirmed the ultrahigh sensitivity of this method for ATM1 up to sub-picomolar concentration in milk samples with a detection limit of 0.3 pg/mL, which allows the method to be used in dairy industry laboratories. Another advantage of our method is that the analysis time is reduced and the sample preparation is very simple and fast in comparison with the conventional methods (HPLC and ELISA, for example). The novel immune-detection platform shows the excellent potential applicability in rapid and sensitive screening of ATM1 toxins in food samples.
